# Real-world analysis of immunochemotherapy in recurrent small-cell lung cancer: opportunities for second-line approaches

**DOI:** 10.3389/fphar.2025.1591643

**Published:** 2025-05-30

**Authors:** Yanrong Guo, Songyan Han, Qinxiang Guo, Jinfang Zhai, Xiaohui Ren, Shengshu Li, Jianchun Duan

**Affiliations:** ^1^ Department of Respiratory Medicine, Shanxi Province Cancer Hospital, Shanxi Hospital Affiliated to Cancer Hospital, Chinese Academy of Medical Sciences, Cancer Hospital Affiliated to Shanxi Medical University, Taiyuan, China; ^2^ Department of Medical Oncology, Cancer Hospital, Chinese Academy of Medical Science and Peking Union Medical College, Beijing, China

**Keywords:** small-cell lung cancer, recurrent, immunochemotherapy, second-line, serplulimab-based

## Abstract

**Background:**

Small-cell lung cancer (SCLC) has limited therapeutic options beyond first-line treatment, and the efficacy of PD-1/PD-L1-based immunotherapy in this setting remains uncertain. This study evaluates the efficacy and safety of serplulimab-based immunochemotherapy as a second- or later-line treatment for SCLC.

**Methods:**

This retrospective, real-world study included 39 SCLC patients treated with post-initial serplulimab-based immunochemotherapy at Shanxi Provincial Cancer Hospital between May 2022 and November 2023. Primary and secondary endpoints were overall survival (OS) and progression-free survival (PFS), respectively. Cox analyses were conducted to explore factors associated with survival outcomes.

**Results:**

The median follow-up duration was 13.7 months. The OS was 12.00 months (95% CI: 6.87-not reached), and the median PFS was 4.07 months (95% CI: 3.07–7.17), with an objective response rate of 20.51%. Patients who underwent immunotherapy re-challenge showed numerically higher median OS (12.77 vs. 9.17 months) and PFS (5.93 vs. 3.87 months) than those without prior immunotherapy. Patients with an objective response to front-line therapy exhibited a trend toward improved median OS (not reached vs. 6.47 months) and PFS (5.93 vs. 3.17 months). Cox analysis identified ECOG PS of 2, elevated LDH, ProGrp, and NSE, and liver metastasis were associated with worse OS. The most common adverse events were thrombocytopenia, elevated ALT, and hypothyroidism, with a manageable safety profile.

**Conclusion:**

Second- or later-line serplulimab-based immunochemotherapy shows promising antitumor activity and survival benefits for SCLC, regardless of prior immunotherapy exposure. Although limited by sample size and retrospective design, these findings highlight the potential of immunotherapy combinations beyond first-line therapy.

## 1 Introduction

Small-cell lung cancer (SCLC) represents one of the most critical oncological challenges worldwide, accounting for approximately 15% of all lung cancer cases (Gazdar et al., 2017). While anti-PD-1/PD-L1-based immunochemotherapy strategies have improved survival outcomes, achieving response rates of 80%–90% in limited-stage SCLC (LS-SCLC) and 50%–80% in extensive-stage SCLC (ES-SCLC) (Schmittel, 2011), this approach remains restricted to first-line settings. Despite initial responses to first-line therapy, median progression-free survival (PFS) is typically less than 6 months (Sathiyapalan et al., 2022; Zhang et al., 2023), and disease progression or recurrence is almost inevitable. Currently, effective options for subsequent lines of therapy remain limited (Tariq et al., 2021). As the standard second-line treatment, topotecan, a topoisomerase I inhibitor, has demonstrated a survival advantage over best supportive care, with a median overall survival (OS) of 25.9 weeks compared to 13.9 weeks (O'Brien et al., 2006). However, its utility is compromised by significant toxicity, primarily myelosuppression and hematologic adverse events, which impact tolerability for many patients (Goto et al., 2016; Das et al., 2021). In 2020, lurbinectedin received conditional approval from the U.S. Food and Drug Administration (FDA) as the first drug in over 20 years for second-line treatment of SCLC, based on an objective response rate (ORR) of 35%. Unfortunately, subsequent randomized trials failed to demonstrate a survival benefit with lurbinectedin in this setting (Trigo et al., 2020; Baena et al., 2021).

The efficacy of PD-1-targeted immune checkpoint inhibitors (ICIs), such as pembrolizumab and nivolumab, in the later-line setting for SCLC remains controversial. In 2020 and 2021, the FDA withdrew accelerated approvals for these agents as third-line options, citing insufficient evidence of survival benefit (Rudin et al., 2020; Owonikoko et al., 2021; Spigel et al., 2021). Despite this, the recent National Comprehensive Cancer Network (NCCN) guidelines recommend PD-1-targeted ICIs as a second-line treatment for patients who have not previously received immunotherapy (NCCN, 2024). Given the significant survival benefit observed with ICIs in the frontline setting (Horn et al., 2018; Paz-Ares et al., 2019; Cheng et al., 2022), further investigation into their potential role in later lines of treatment is warranted to address the unmet needs in SCLC management.

Serplulimab, an anti-PD-1 monoclonal antibody, has demonstrated promising efficacy in the international phase III ASTRUM-005 trial, showing a median OS benefit of 15.4 months compared to 10.9 months in ES-SCLC patients (Cheng et al., 2022). Based on these results, regulatory authorities such as China’s National Medical Products Administration (NMPA), the Indonesian Food and Drug Authority (BPOM), and the European Medicines Agency (EMA) have approved serplulimab in combination with etoposide and platinum as a first-line treatment for ES-SCLC. Several studies have highlighted its therapeutic potential and its cost-effectiveness advantage, particularly for Chinese patients (Shao et al., 2023; Liu et al., 2024). In the absence of alternative options for recurrent SCLC, clinicians may still consider serplulimab-based immunotherapy or re-challenge with immunotherapy. This study aims to leverage real-world data from a single-center retrospective cohort to evaluate the efficacy and safety of serplulimab-based regimens in SCLC patients beyond first-line treatment.

## 2 Subjects and methods

### 2.1 Patients

This retrospective, real-world study was conducted at the Shanxi Provincial Cancer Hospital, with medical records of patients reviewed by investigators between May 2022 and November 2023. The inclusion criteria were: (1) age ≥ 18 years old; (2) histologically or cytologically confirmed SCLC; (3) disease progression or recurrence after at least one prior regimen; (4) treatment with either a serplulimab-based combination or serplulimab monotherapy as second- or later-line therapy. The exclusion criteria were: (1) insufficient clinical data and (2) the presence of other primary malignancies. This study was approved by the Ethics Committee of the Shanxi Provincial Cancer Hospital (No. KY2024046), with a waiver of written informed consent from patients, and was conducted in accordance with the Declaration of Helsinki. This study was reported following the Strengthening the Reporting of Observational Studies in Epidemiology (STROBE) guidelines (Supplementary Material).

### 2.2 Data collection and outcome assessment

We gathered a spectrum of demographic and clinicopathological characteristics, including age, sex, body mass index (BMI), smoking status, family tumor history, Eastern Cooperative Oncology Group performance status (ECOG PS), clinical stage, metastasis status, treatment regimens, biomarkers, and toxicity. The antitumor activity of the first line was also collected. Clinical data were collected from the electronic medical database and telephone follow-ups.

The primary endpoint of this study was OS, defined as the time from the first dose of serplulimab-based second- or later-line treatment to death from any cause. The secondary endpoint was PFS, defined as the time from serplulimab-based second or further-line treatment to the first documented disease progression or death from any cause. We also performed subgroup analyses to explore clinicopathological factors that may be associated with treatment efficacy. Additionally, the ORR was calculated as the proportion of patients who achieved a complete response (CR) or partial response (PR) according to the Response Evaluation Criteria in Solid Tumors version 1.1 (RECIST v1.1). The disease control rate (DCR) was calculated as the proportion of patients achieving CR, PR, or stable disease (SD) for at least 4 weeks. Adverse events (AEs) during serplulimab-based treatment were assessed according to the Common Terminology Criteria for Adverse Events (CTCAE) version 5.0 by two independent investigators who reviewed safety events recorded in medical charts. Any discrepancies were resolved by the third investigator.

### 2.3 Statistical analysis

Categorical variables were summarized as counts and percentages, while continuous variables were described using mean ± standard deviation or median (range), as appropriate. PFS and OS were estimated with the Kaplan-Meier method, and median values with 95% confidence intervals (CIs) were calculated. Survival and tumor response analyses were further stratified based on first-line tumor response status and prior immunotherapy exposure. To identify survival risk factors, univariate and multivariate Cox proportional-hazards models were applied, with hazard ratios (HR) and corresponding 95% CIs reported. Data management and statistical analyses were performed using R software (version 4.3.2), with statistical significance defined at a two-sided *P*-value of < 0.05.

## 3 Results

### 3.1 Baseline characteristics

**FIGURE 1:**
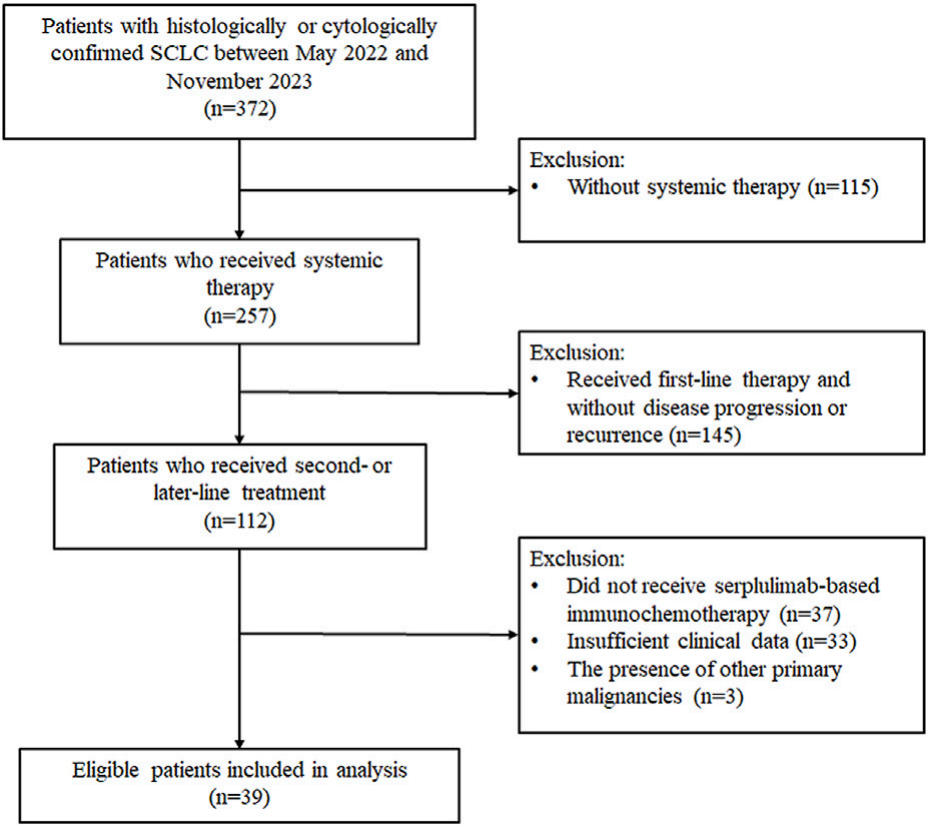
Flowchart of patients screening. SCLC, small-cell lung cancer.

We reviewed the medical records of 372 SCLC patients treated at the Shanxi Provincial Cancer Hospital between May 2022 and November 2023. Among them, 257 patients received systemic therapy, and 112 had documented second- or later-line treatment. After applying the inclusion and exclusion criteria, 39 patients were included in this retrospective analysis (Figure 1). Most of the included patients (n = 33, 84.62%) received second-line serplulimab-based therapy. The baseline characteristics of these 39 patients are shown in Table 1. The mean age was 56.56 years, with the majority being male (87.18%), smokers (79.49%), having an ECOG PS of 1 (92.31%), and presenting with stage IV disease (69.23%). Sixteen patients (41.03%) had received prior immunotherapy, and 26 patients (66.67%) achieved an objective response to first-line therapy. The majority received immunochemotherapy beyond first-line with taxane- (n = 22, 56.41%) or platinum-based (n = 27, 69.23%) regimens, with 13 of them receiving both taxane- and platinum-based chemotherapy. Additionally, another three patients received serplulimab combined with anlotinib (n = 1), vinorelbine (n = 1), and temozolomide (n = 1), respectively.

**TABLE 1:** Baseline characteristics of included patients.

Characteristics, n (%)	Patients
	(N = 39)
Age^a^, y	56.56 ± 10.39
Age	
<60 years	21 (53.85)
≥60 years	18 (46.15)
Gender	
Male	34 (87.18)
Female	5 (12.82)
BMI	
<18.5 kg/m^2^	2 (5.13)
18.5–24.9 kg/m^2^	19 (48.72)
≥25 kg/m^2^	18 (46.15)
Family tumor history	
Yes	6 (15.38)
No	33 (84.62)
History of smoking	
Yes	31 (79.49)
No	8 (20.51)
ECOG PS	
1	36 (92.31)
2	3 (7.69)
History of complication	
Yes	11 (28.21)
No	28 (71.79)
Clinical stage	
Ⅲ	12 (30.77)
Ⅳ	27 (69.23)
Baseline NLR	
<3	13 (33.33)
≥3	26 (66.67)
Baseline LDH	
<225 U/I	21 (53.85)
≥225 U/I	18 (46.15)
Baseline ProGrp	
<300 ng/L	30 (76.92)
≥300 ng/L	9 (23.08)
Baseline NSE	
≤16.3 ng/mL	37 (94.87)
>16.3 ng/mL	2 (5.13)
Baseline CEA	
<6 ng/mL	35 (89.74)
≥6 ng/mL	4 (10.26)
Bone metastasis	
Yes	5 (12.82)
No	34 (87.18)
Brain metastasis	
Yes	13 (33.33)
No	26 (66.67)
Liver metastasis	
Yes	7 (17.95)
No	32 (82.05)
Treatment lines	
2	33 (84.62)
≥3	6 (15.38)
Combined chemotherapy regimen^b^	
Taxane-based	22 (56.41)
Platinum-based	27 (69.23)
Others^c^	3 (7.69)
Prior immunotherapy	
Yes	16 (41.03)
No	23 (58.97)
First-line SD/PD	
Yes	13 (33.33)
No	26 (66.67)

^a^ data was presented as mean ± standard deviation.

^b^ Thirteen patients received both taxane- and platinum-based as the chemotherapy regimen, resulting in an overall percentage greater than 100.

^c^ Patients received anlotinib (n = 1), vinorelbine (n = 1), and temozolomide (n = 1) chemotherapy.

BMI, body mass index; ECOG PS, eastern cooperative oncology group performance status; NLR, neutrophil-lymphocyte ratio; LDH, lactate dehydrogenase; ProGrp, Pro Gastrin-Releasing Peptide; NSE, Neuron-Specific Enolase; CEA, carcinoembryonic antigen; SD, stable disease; PD, progressive disease.

### 3.2 Efficacy and subgroup analysis

**FIGURE 2:**
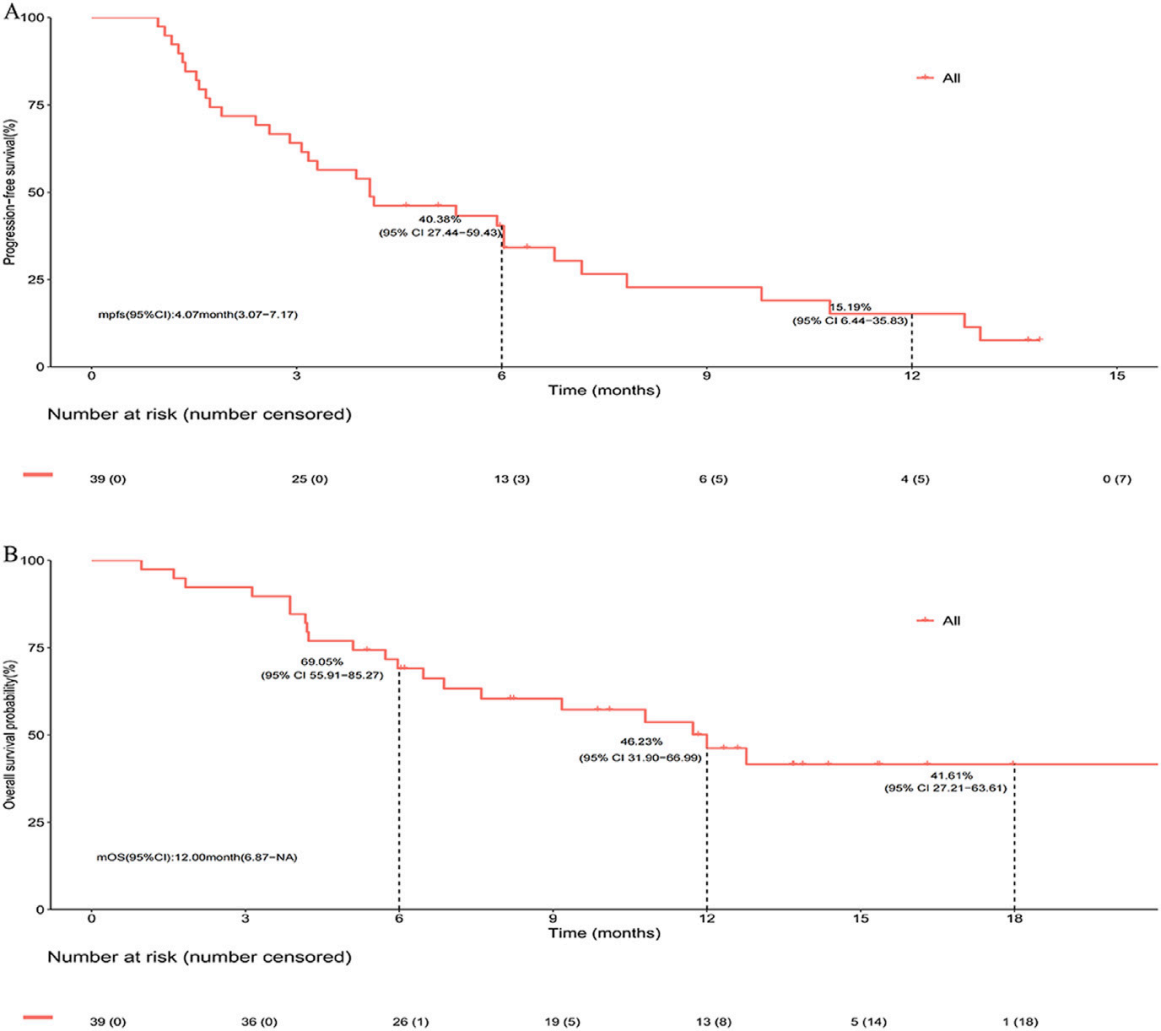
Survival of entire cohort following second- or later-line serplulimab-based immunochemotherapy. **(A)** Kaplan-Meier estimates of progress-free survival. **(B)** Kaplan-Meier estimates of overall survival.

**FIGURE 3:**
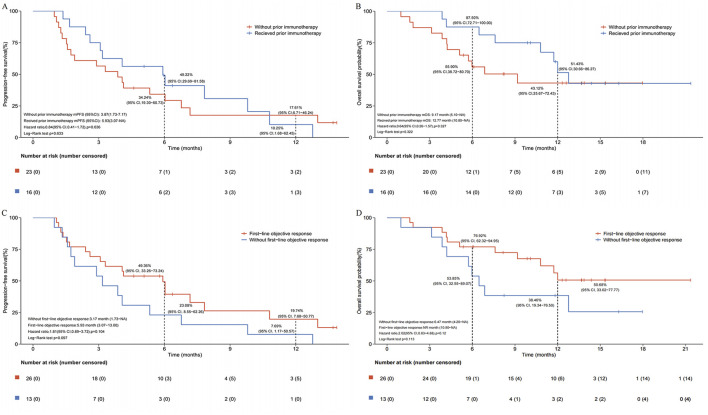
Subgroup analysis of survival following second- or later-line serplulimab-based immunochemotherapy. **(A)** Kaplan-Meier estimates of progress-free survival stratified by prior immunotherapy exposure. **(B)** Kaplan-Meier estimates of overall survival stratified by prior immunotherapy exposure. **(C)** Kaplan-Meier estimates of progress-free survival stratified by first-line tumor response status. **(D)** Kaplan-Meier estimates of overall survival stratified by first-line tumor response status.

After a median follow-up of 13.7 months, the median PFS (mPFS) and median OS (mOS) for the entire cohort were 4.07 months (95% CI, 3.07–7.17) and 12.00 months (95% CI, 6.87-not reached), respectively (Figures 2A,B). In subgroup analysis stratified by prior immunotherapy, patients who underwent immunotherapy re-challenge showed numerically higher mPFS and mOS than those without prior immunotherapy, although the differences were not statistically significant (mPFS: 5.93 vs. 3.87 months, log-rank *P* = 0.633; mOS: 12.77 vs. 9.17 months, log-rank *P* = 0.322) (Figures 3A,B). Univariate Cox analysis also confirmed no significant association between prior ICIs use and survival outcomes in subsequent immunotherapy (both *P* > 0.05, Table 2). Patients with an objective response to front-line therapy had numerically longer mPFS (5.93 vs. 3.17 months, log-rank *P* = 0.097, Figure 3C) and mOS (not reached vs. 6.47 months, log-rank *P* = 0.113, Figure 3D) compared to those without an objective response. Univariate Cox analysis also showed that the relationship between first-line tumor response status and survival was not statistically significant (both *P* > 0.05, Table 2).

**TABLE 2:** Univariate and multivariate Cox analyses of PFS and OS for SCLC patients following second or later-line serplulimab-based immunochemotherapy.

Variable	PFS				OS			
	Univariate analysis		Multivariate analysis		Univariate analysis		Multivariate analysis	
	HR (95%CI)	*P*	HR (95%CI)	*P*	HR (95%CI)	*P*	HR (95%CI)	*P*
Gender*								
Male	Ref.		Ref.					
Female	0.14 (0.03–0.61)	0.009	0.19 (0.04–0.86)	0.031				
Age								
<60 years	Ref.				Ref.			
≥60 years	1.09 (0.54–2.18)	0.814			1.86 (0.76–4.56)	0.175		
BMI								
<18.5 kg/m^2^	Ref.				Ref.			
18.5–24.9 kg/m^2^	0.57 (0.13–2.52)	0.459			0.46 (0.10–2.14)	0.325		
≥25 kg/m^2^	0.53 (0.12–2.35)	0.400			0.38 (0.08–1.8)	0.221		
Family tumor history								
No	Ref.		Ref.		Ref.			
Yes	2.90 (1.12–7.47)	0.028	3.54 (1.26–9.98)	0.017	2.18 (0.72–6.62)	0.168		
History of smoking								
No	Ref.				Ref.			
Yes	1.74 (0.70–4.34)	0.233			1.55 (0.45–5.31)	0.482		
ECOG PS								
1	Ref.		Ref.		Ref.		Ref.	
2	7.22 (1.85–28.25)	0.004	4.35 (0.59–32.31)	0.151	5.24 (1.40–19.58)	0.014	5.10 (0.62–41.70)	0.129
History of complication								
No	Ref.				Ref.			
Yes	0.54 (0.23–1.25)	0.148			0.49 (0.16–1.49)	0.210		
Clinical stage								
Ⅲ	Ref.				Ref.			
Ⅳ	1.76 (0.77–3.99)	0.178			2.13 (0.71–6.38)	0.176		
Baseline NLR								
<3	Ref.				Ref.			
≥3	1.26 (0.59–2.69)	0.552			0.64 (0.26–1.56)	0.325		
Baseline LDH								
<225 U/I	Ref.		Ref.		Ref.		Ref.	
≥225 U/I	2.22 (1.10–4.49)	0.026	1.57 (0.66–3.72)	0.305	3.80 (1.45–9.97)	0.007	2.61 (0.86–7.90)	0.090
Baseline ProGrp								
<300 ng/L	Ref.		Ref.		Ref.		Ref.	
≥300 ng/L	3.69 (1.60–8.48)	0.002	2.21 (0.80–6.14)	0.128	5.66 (2.22–14.38)	<0.001	1.89 (0.55–6.50)	0.312
Baseline NSE								
≤16.3 ng/mL	Ref.		Ref.		Ref.		Ref.	
>16.3 ng/mL	6.58 (1.38–31.29)	0.018	1.10 (0.10–11.67)	0.936	5.60 (1.22–25.73)	0.027	0.65 (0.07–6.51)	0.715
Baseline CEA								
<6 ng/mL	Ref.				Ref.			
≥6 ng/mL	1.28 (0.44–3.68)	0.652			1.34 (0.31–5.83)	0.693		
Bone metastasis								
No	Ref.				Ref.			
Yes	1.45 (0.50–4.21)	0.496			1.72 (0.50–5.92)	0.386		
Brain metastasis								
No	Ref.				Ref.			
Yes	1.08 (0.51–2.26)	0.848			1.00 (0.40–2.52)	0.995		
Liver metastasis								
No	Ref.				Ref.		Ref.	
Yes	2.15 (0.85–5.44)	0.106			3.65 (1.39–9.58)	0.008	2.77 (0.85–9.06)	0.091
Prior immunotherapy								
No	Ref.				Ref.			
Yes	0.84 (0.41–1.72)	0.636			0.64 (0.26–1.57)	0.327		
First-line SD/PD								
No	Ref.				Ref.			
Yes	1.81 (0.89–3.72)	0.104			2.02 (0.83–4.88)	0.120		

* The relationship between gender and OS, was not analyzed because the model did not converge due to the absence of mortality events in female patients.

PFS, progress-free survival; OS, overall survival; HR, hazard ratio; BMI, body mass index; ECOG PS, eastern cooperative oncology group performance status; NLR, neutrophil-to-lymphocyte ratio; LDH, lactate dehydrogenase; ProGrp, pro-gastrin-releasing peptide; NSE, neuron-specific enolase; CEA, carcinoembryonic antigen; SD, stable disease; PD, progressive disease.

During immunochemotherapy beyond first-line, 8/39 patients achieved an objective response (all PR), 17/39 achieved SD, and 14/39 had progressive disease. Overall, the ORR was 20.51% (8/39; 95% CI, 9.30–36.46) and DCR was 64.10% (25/39; 95% CI, 47.18–78.80). Additionally, patients with prior immunotherapy showed numerically higher ORR (31.25% vs. 13.04%) and DCR (75.00% vs. 56.52%) compared to those without prior immunotherapy.

Based on clinicopathological characteristics, we conducted an exploratory analysis of factors related to PFS and OS in patients receiving immunochemotherapy beyond first-line using a Cox proportional hazards model (Table 2). Univariate analysis for PFS indicated that females were associated with significantly better PFS (HR, 0.14; 95% CI, 0.03–0.61; *P* = 0.009). In contrast, factors associated with poorer PFS included family history of cancer, ECOG PS of 2, elevated baseline levels of lactate dehydrogenase (LDH), pro-gastrin-releasing peptide (ProGrP), and neuron-specific enolase (NSE) (all *P* < 0.05). On multivariate analysis, the association of PFS with gender and family history of cancer was also confirmed (all *P* < 0.05). For OS, only univariate analysis showed that ECOG PS of 2, elevated baseline levels of LDH, ProGrp, and NSE, and the presence of liver metastasis were associated with worse OS (all *P* < 0.05).

### 3.3 Safety

Through a comprehensive review of medical records and telephone follow-ups, we identified 47 AEs in 24 patients, resulting in an overall AE incidence of 61.54%. The incidence of grade 1–2 AEs and grade ≥3 AEs were 46.15% and 5.13%, respectively. Additionally, there were 12 AEs in eight patients for which severity could not be assessed due to incomplete documentation and patient refusal to provide further information. The most commonly reported AEs included thrombocytopenia, elevated alanine aminotransferase (ALT), and hypothyroidism (Table 3). All patients tolerated the combination immunotherapy well, and no treatment-related deaths were reported.

**TABLE 3:** AEs of SCLC patients following second or later-line serplulimab-based immunochemotherapy.

AEs, n (%)	Total	Grade 1–2	Grade 3–4
Total	24 (61.54)	18 (46.15)	2 (5.13)
Incidence ≥ 5%			
Thrombocytopenia	9 (23.08)	8 (20.51)	1 (2.56)
Elevated ALT	6 (15.38)	5 (12.82)	‾
Hypothyroidism	6 (15.38)	2 (5.13)	‾
Anemia	5 (12.82)	5 (12.82)	‾
Elevated AST	4 (10.26)	2 (5.13)	‾
Neutropenia	4 (10.26)	3 (7.69)	1 (2.56)
Pneumonia	2 (5.13)	2 (5.13)	‾
Myelosuppression	2 (5.13)	2 (5.13)	‾

AEs, adverse events; ALT, alanine aminotransferase; AST, aspartate aminotransferase.

## 4 Discussion

In this retrospective, real-world study, we evaluated the efficacy of serplulimab-based immunochemotherapy as second- or later-line therapy for patients with SCLC. Our results suggest that this treatment regimen had impressive antitumor activity and encouraging survival outcomes, with 20.51% of patients achieving an ORR, a mPFS of 4.07 months, and a mOS of 12.00 months. Additionally, patients who had received prior immunotherapy and achieved an objective response in front-line therapy showed improved antitumor activity and survival outcomes with serplulimab-based later-line therapy. Most patients (66.67%) included in our study achieved an objective response to first-line therapy, which is consistent with the approximately 70% reported in previous studies (Paz-Ares et al., 2019; Rudin et al., 2020). This consistency enhances the credibility of our findings, even with the limitation of the small sample size included in our cohort.

The role of PD-1/PD-L1-based immunotherapy in second-line treatment for SCLC has been explored in the CheckMate 331 trial (Spigel et al., 2021). In this study, nivolumab monotherapy did not demonstrate an OS benefit, with a median OS of 7.5 months compared to 8.4 months in the topotecan or amrubicin chemotherapy group (HR, 0.86; 95% CI, 0.72–1.04; *P* = 0.11). Similarly, mPFS showed no advantage with nivolumab monotherapy (1.4 vs. 3.8 months; HR, 1.41; 95% CI, 1.18–1.69). These results suggest that mono-immunotherapy offers limited efficacy for previously treated SCLC patients. However, the trial identified subgroups with low LDH levels and no liver metastases as having better survival benefits from immunotherapy, which is consistent with our findings. Nivolumab also showed limited efficacy as a third-line therapy in SCLC, with the CheckMate 032 trial reporting a median OS of 5.6 months and a median PFS of 1.4 months (Ready et al., 2019). In addition, a pooled analysis of the phase Ib KEYNOTE-028 and phase II KEYNOTE-158 studies showed that pembrolizumab achieved an mOS of 7.7 months and an mPFS of 2.0 months in patients with recurrent or metastatic SCLC who had received two or more prior therapies (Chung et al., 2020). In our study, all patients received serplulimab-based combination regimens, which may have contributed to the relatively longer survival observed in our cohort. This finding aligns with previous reports demonstrating that immunotherapy-chemotherapy combinations achieved median PFS ranging from 3.2 to 4.8 months in patients with previously treated SCLC (Ishii et al., 2021; Liu et al., 2025). The therapeutic synergy likely stems from checkpoint blockade-enhanced T-cell cytotoxicity coupled with chemotherapy-induced immunogenic cell death, which promotes tumor antigen release and dendritic cell maturation. Lurbinectedin, a newly FDA-approved second-line option, is a synthetic alkaloid that covalently binds to DNA, inducing cell death. In a phase II study of lurbinectedin for second-line SCLC, an ORR of 35% and a median duration of response (mDOR) of 5.3 months were observed (Trigo et al., 2020). Additionally, tarlatamab (AMG 757), a bispecific T-cell engager molecule targeting delta-like ligand 3 (DLL3) and CD3, demonstrated preliminary efficacy and safety in recurrent SCLC, with an ORR of 23.4%, median PFS of 3.7 months, and median OS of 13.2 months (Paz-Ares et al., 2023). However, the journey from developing a novel therapy to regulatory approval and clinical application is lengthy and complex. Thus, alongside ongoing drug development, there is a critical need to enhance patient benefits using existing agents. Our study suggests that the immunotherapy-based combination strategies may provide additional clinical benefits for SCLC patients beyond first-line treatment.

Although not statistically significant, our data showed a trend toward improved OS in patients who received immunotherapy rechallenge (12.77 vs. 9.17 months), consistent with previous studies. Campelo et al. (García-Campelo et al., 2023) reported potential survival benefits with atezolizumab rechallenge in patients with progressed ES-SCLC. Other studies have shown that rechallenge with immunochemotherapy can achieve durable antitumor activity and significant survival benefits compared to monotherapy approaches (Li et al., 2022; Zhang et al., 2024). For our treatment regimen, previous research has indicated that serplulimab combined with chemotherapy may provide additive and synergistic effects, reaffirming immunochemotherapy as a viable strategy in both first-line and subsequent lines for SCLC patients (Kataoka et al., 2020; Ishii et al., 2021). Additionally, our study revealed an interesting phenomenon: patients who achieved tumor response in front-line therapy continued to benefit from subsequent immunotherapy. This result may be attributed to several factors (Tang et al., 2016; Xia et al., 2024). First, the initial tumor response may successfully activate the immune microenvironment, allowing for a more robust response to subsequent immunotherapy. Second, the reduction in tumor burden and reshaping of the tumor microenvironment following front-line therapy may enhance the efficacy of subsequent immunotherapy.

Our Cox analysis revealed a significant association between the female gender and longer PFS in the recurrent SCLC setting, indicating that female patients might experience enhanced benefits from immunotherapy. While the correlation between gender and prognosis in SCLC remains inconsistent, most studies suggesting better survival were related to the female gender and largely due to the fewer smokers (Lim et al., 2018; Tas et al., 2024). However, our findings support potential biological differences in immune response between genders. Factors such as sex hormones, genetic polymorphisms, and immune modulation may contribute to these observed differences, and potentially influence the immunotherapy efficacy (Salgado et al., 2015; Lim et al., 2018; Vavala et al., 2021). In addition to LDH, elevated levels of ProGRP and NSE were associated with worse outcomes in our cohort. These markers, which are associated with neuroendocrine differentiation in SCLC, are generally associated with tumor aggressiveness and worse prognosis, consistent with previous research suggesting that they may indicate reduced tumor responsiveness and poorer patient survival outcomes (Shibayama et al., 2001; Li et al., 2023; Muley et al., 2024). Notably, despite extensive biomarker exploration in SCLC, no validated predictive biomarker has emerged to reliably identify patient subsets benefiting from PD-L1/PD-1 inhibitor-based regimens. While PD-L1 expression serves as a key biomarker for immunotherapy selection in non-small cell lung cancer (NSCLC), its predictive utility has not been reliably established in SCLC. For instance, pooled analysis of KEYNOTE-158 and KEYNOTE-028 trials revealed that pembrolizumab exhibited antitumor activity in heavily pretreated SCLC patients regardless of PD-L1 expression status (Chung et al., 2020). Similarly, the CheckMate 331 trial showed no survival benefit of nivolumab over chemotherapy in relapsed SCLC when stratified by PD-L1 combined positive score at a cutoff of 1% (Spigel et al., 2021). The use of tumor mutational burden (TMB) in SCLC also yielded inconclusive results. A correlation between TMB and tumor response was observed in the CheckMate 032 trial using whole-exome sequencing, but this association was not replicated in the IMpower 133 trial where circulating tumor DNA analysis was employed (Horn et al., 2018; Ready et al., 2020).

As noted, our study is limited by its sample size and geographic scope, which introduces the potential for selection bias and limits the generalizability of our findings to the broader SCLC population. Additionally, as a single-cohort retrospective analysis, our study lacks a control group, restricting us to comparisons with historical data rather than allowing for direct, controlled comparisons with other second- or later-line therapies. This study design further limits our ability to establish causative relationships between treatment regimens and outcomes. The undocumented severity of 12 adverse events in eight patients, attributable to incomplete medical records or patient refusal, may underestimate toxicity risks, underscoring the necessity for enhanced real-time monitoring and standardized reporting in future studies in the real-world research. Moreover, due to the absence of an in-depth biomarker analysis, we were unable to investigate the underlying mechanisms by which patients may benefit from serplulimab-based combination therapy. Future multicenter, prospective studies with larger, more diverse cohorts and biomarker evaluation are essential to validate these findings and to explore potential mechanisms driving response.

In conclusion, this real-world study suggests that immunochemotherapy as a second- or later-line treatment demonstrates promising efficacy and safety in SCLC patients, regardless of prior immunotherapy exposure and first-line tumor response status. Although limited by sample size and study design, the tendency towards extended survival in patients rechallenged with immunotherapy reaffirms immunochemotherapy as a feasible approach for SCLC patients in both first-line and subsequent lines. Our findings underscore the need for further investigation into tailored immunotherapy approaches that could maximize clinical benefit in SCLC, supporting the rationale for immunochemotherapy beyond first-line treatment.

## Data Availability

The original contributions presented in the study are included in the article/Supplementary Material, further inquiries can be directed to the corresponding author.
